# Redefining Cheminformatics with Intuitive Collaborative Mobile Apps

**DOI:** 10.1002/minf.201200010

**Published:** 2012-07-04

**Authors:** Alex M Clark, Sean Ekins, Antony J Williams

**Affiliations:** a Molecular Materials Informatics, 1900 St. Jacques #302, Montreal, Quebec, Canada H3J 2S1; b Collaborations in Chemistry, 5616 Hilltop Needmore Road, Fuquay Varina, NC 27526, USA; c Royal Society of Chemistry, 904 Tamaras Circle, Wake Forest, NC-27587, USA

**Keywords:** Apps, Chemistry, Collaboration, ChemSpider, Cloud Computing, Mobile Chemistry, Mobile Reagents, Web Services

## Abstract

The proliferation of mobile devices such as smartphones and tablet computers has recently been extended to include a growing ecosystem of increasingly sophisticated chemistry software packages, commonly known as apps. The capabilities that these apps can offer to the practicing chemist are approaching those of conventional desktop-based software, but apps tend to be focused on a relatively small range of tasks. To overcome this, chemistry apps must be able to seamlessly transfer data to other apps, and through the network to other devices, as well as to other platforms, such as desktops and servers, using documented file formats and protocols whenever possible. This article describes the development and state of the art with regard to chemistry-aware apps that make use of facile data interchange, and some of the scenarios in which these apps can be inserted into a chemical information workflow to increase productivity. A selection of contemporary apps is used to demonstrate their relevance to pharmaceutical research. Mobile apps represent a novel approach for delivery of cheminformatics tools to chemists and other scientists, and indications suggest that mobile devices represent a disruptive technology for drug discovery, as they have been to many other industries.

## 1 Introduction

A recent perspective suggested that the field of cheminformatics is still addressing fundamental problems that have existed for decades, rather than focusing on innovation.^1^ If anything, it has not perhaps brought about any major new breakthroughs since the golden age of the late 1980s and early 1990s during which techniques such as Comparative Molecular Field Analysis (CoMFA),^2^ ligand-protein docking,^3^ pharmacophore searching,^4^–^6^ NMR prediction,^7^,^8^ and nomenclature generation^9^ were developed, among others.^10^–^12^ The authors suggested that the few major companies in this field developed me-too technologies, and are therefore ripe for disruption. One area of interest is collaboration and systems for secure but selective sharing of chemical information^13^ between groups. We feel that this is particularly important as major pharmaceutical, chemical and consumer product organizations turn to open innovation, crowdsourcing, outsourcing, alliances and other forms of collaboration in order to innovate more quickly and cost effectively.^14^–^16^ As these various industries are under different pressures (e.g. shareholder, regulatory, environmental, etc.) to improve by either increasing productivity, making cleaner products, reducing animal testing, etc., this places software providers in the position of needing to adapt quickly to the new requirements. There is a shift toward using more predictive technologies to estimate risk (ADME/Tox, environmental impact etc.)^17^ and the users of such models and tools may be global and potentially involve collaboration between different companies or public private partnerships (e.g. eTox and OpenTox). In many cases contract research organisations may be collaborating with their clients forming truly complex networks in which there will need to be combinations of openness, selective sharing and complete privacy.^18^,^19^ We are also witnessing an increased role for pre-competitive initiatives^16^ to prevent duplication of efforts and to facilitate cost and risk sharing (e.g. Pistoia Alliance^20^ and Open PHACTS^21^). Another disruptive pressure for cheminformatics is coming from the various open source toolkits and initiatives for model building, descriptors and model hosting.^22^–^35^

It is against this backdrop that the user community is demanding a new breed of chemical information software that keeps pace with the rapidly changing dynamics within the chemical industry in general, and pharmaceuticals in particular. The era of expensive per-seat licensing for monolithic software with a steep learning curve is drawing to a close. Software for chemists has to be affordable enough for all chemists to participate, have a sufficiently intuitive user interface that becoming an expert is not mandatory, and be available anywhere, anytime. We have already seen a significant amount of effort expended in an attempt to commoditize desktop software, to provide web interfaces, and to move server-side functionality into the cloud. We have also seen an increasing amount of formerly expensive software challenged by competent open source alternatives.^36^–^39^

Already the hardware and operating environment of a contemporary smartphone or tablet computer is capable of delivering a large fraction of the functionality that was once the exclusive domain of desktop workstations. By making optimal use of the limited, though still impressive, computational and graphics capabilities of the device, and delegating key resource-intensive activities to web services, a considerable amount of work can be carried out on a device that weighs less than a small book, and can be used in just about any environment.^40^,^41^ We are now seeing and participating in the development of a chemistry app ecosystem, which has grown from a handful of crude apps two years ago, into a range of powerful tools. These first generation chemistry apps enable tasks such as molecule sketching, reaction drawing, data entry, structure searching, portals to other software and databases, educational tools, chemistry flashcard tests, games, puzzles, and access to scientific content from publishers.^42^ Some of these apps are multifunctional, while others are narrow in scope, but no single app provides all of the necessary tools for every chemist. Due to their very modular nature, and their tendency to rely on interaction with network resources, data sharing is of paramount importance to the development of a mobile app ecosystem for chemistry. If each of the apps performed just its own function, in isolation, the opportunities for these devices would be very limited. To realize its full potential, a chemistry app must be able to interoperate with other apps in a complementary fashion, and it must also be able to pass data back and forth between a group of collaborators with heterogeneous technology platforms, using standard data formats and transport mechanisms whenever possible. Mobile chemistry apps that fully embrace interoperability with desktop and server based software, as well as other apps and internet resources, will have a more prominent future ahead of them because of their capability to be useful for extended workflows as seen in pharmaceutical research.^39^–^41^

## 2 Methods

### 2.1 Mobile Devices and Data Sharing

Contemporary mobile devices use operating systems originally designed for smartphones (e.g. iPhone OS, Android, BlackBerry OS, Windows Phone, WebOS, etc.). Data communication is based on mature technology which is integrated into each device, whether it be a phone, tablet or portable music player. We believe these devices now represent considerable computing power sufficient for them to be viable tools for chemistry leveraging cheminformatics. The capabilities of interest for chemical collaboration using mobile apps can be roughly divided into 3 categories

– Networked communication of chemical data, including accessing data in online databases.
– App-to-app data transmission of data.
– Preparation of presentation graphics that include chemistry-related content, namely molecules or reactions.

The networked communication of chemical data using standard formats is the single most important capability when it comes to incorporating mobile apps within a cheminformatics workflow. Since most of the tasks for which chemists use software are carried out using some combination of workstations, servers and sometimes multiple mobile devices, the interchange of data between platforms must be effective and seamless.

One of the most generally applicable techniques for communication is the use of email attachments. An email attachment consists of a filename, a MIME type,^43^ and arbitrary text or binary data. By using chemical data formats that are publicly documented, attachments can be identified by the file extension or MIME type. Interoperability between workstation and mobile software can be accomplished simply by sending an email which includes the appropriate data, in the form an attachment, or multiple attachments encoding the same data in different formats, as long as at least one of the formats is recognized by the receiving software. By making available the most commonly implemented lowest-common-denominator formats, such as the MDL Molfile or MDL SDfile^44^ it is possible to ensure that the largest possible variety of cheminformatics software is capable of participating in data exchange.

For the sending or receiving of emails using software designed for workstations it is common to use a software package to prepare an attachment as a structure file then send it via email. For inbound content, the attachment is saved as a file and opened with the appropriate software package. For mobile apps, the data is typically packaged or unpackaged directly by the app itself using a transport mechanism provided by the operating system.

All modern mobile devices feature a powerful web browser. When encountering chemical data during mobile browsing, the handling mechanism is very similar to dealing with incoming email attachments: the file type is identified by its file extension and MIME type, and the mobile operating system is able to delegate to any app that has registered itself as being able to handle the attachment. Thus, browsing online data repositories is an effective form of one-way communication for loading data onto a mobile device.

Being able to instantiate HTTP connections allows mobile devices to access an unlimited variety of web service functionality. These *remote procedure calls* are even more important to mobile apps than to workstation-based software, since they often involve consulting a centralized chemical database, or providing access to computational features that cannot readily be adapted to run on the mobile device. This data transport mechanism can also be used to provide app-generated content to a remote service, or when implemented in a bidirectional capacity, can be used to implement remote file hosting (e.g. accessing a file sharing service such as Dropbox,^45^ discussed in detail below).

Cheminformatics software packages designed for workstations typically operate on large or entire portions of a workflow, with importing or exporting of data being a concern only at the beginning or the end. The modular nature of mobile apps means that it is often necessary to use more than one app to accomplish a particular workflow segment, e.g. using a database searching app to locate data, and another to organize it into a collection. Passing data back and forth between apps is therefore an integral and frequent activity. One reliable technique is to use the system-wide clipboard, which allows arbitrary text content to be placed in a repository that is accessible to any other app, on demand. The *copy*-and-*paste* sequence is a well-established user interface paradigm, and works the same way on mobile devices as it does for workstations. Since most chemical data formats are text-based, they can easily be encoded onto the clipboard. When an app is instructed to *paste* the content, it attempts to parse the data, and if successful, can undertake a context-specific action, e.g. adding a molecule to the current collection.

Mobile operating systems also provide a variety of less manual techniques for *interprocess communication*, which allows one app to address another app and pass data to it directly. There are multiple techniques available, some of which are discussed subsequently. The ability for apps to address each other generally or specifically, and pass messages that include chemical data, allows the creation of a toolbox of apps, which can be combined together to accomplish larger tasks.

### 2.2 Application of Apps to a Chemistry Workflow

While a cheminformatics workflow often has objectives such as gaining an understanding of an aspect of chemistry or biochemistry, or accumulating valuable data in a repository, a very large part of the value proposition of software for chemists is the ability to create publication-quality graphics of structures and data. Trained chemists can readily interpret a visual depiction of a chemical. It is now possible to create high quality graphics on a mobile device, either directly from within an app itself, or by making use of a remote procedure call. Graphics can be made available to other apps installed on the same device, e.g. by placing images onto the clipboard, printing, or exporting via network transport techniques, such as email or remote file hosting. In this way, chemical graphics created using mobile devices can be incorporated into other software, such as office productivity packages that are typically used for preparing manuscripts, slideshow presentations or websites.

The remainder of this section describes implementation details for the key data-sharing technologies described in this article, using existing apps as illustrative examples. The discussion is largely limited to apps designed for the Apple iOS platform, which includes iPhones, iPods and iPads, simply because the proliferation of chemistry apps for iOS is far more advanced at the time of writing. The technologies described generalize well to other contemporary platforms, such as Android, BlackBerry OS and Windows Phone, and it is expected that analogous chemistry software will be ported to these platforms as they gain in popularity.

**Figure 1:**
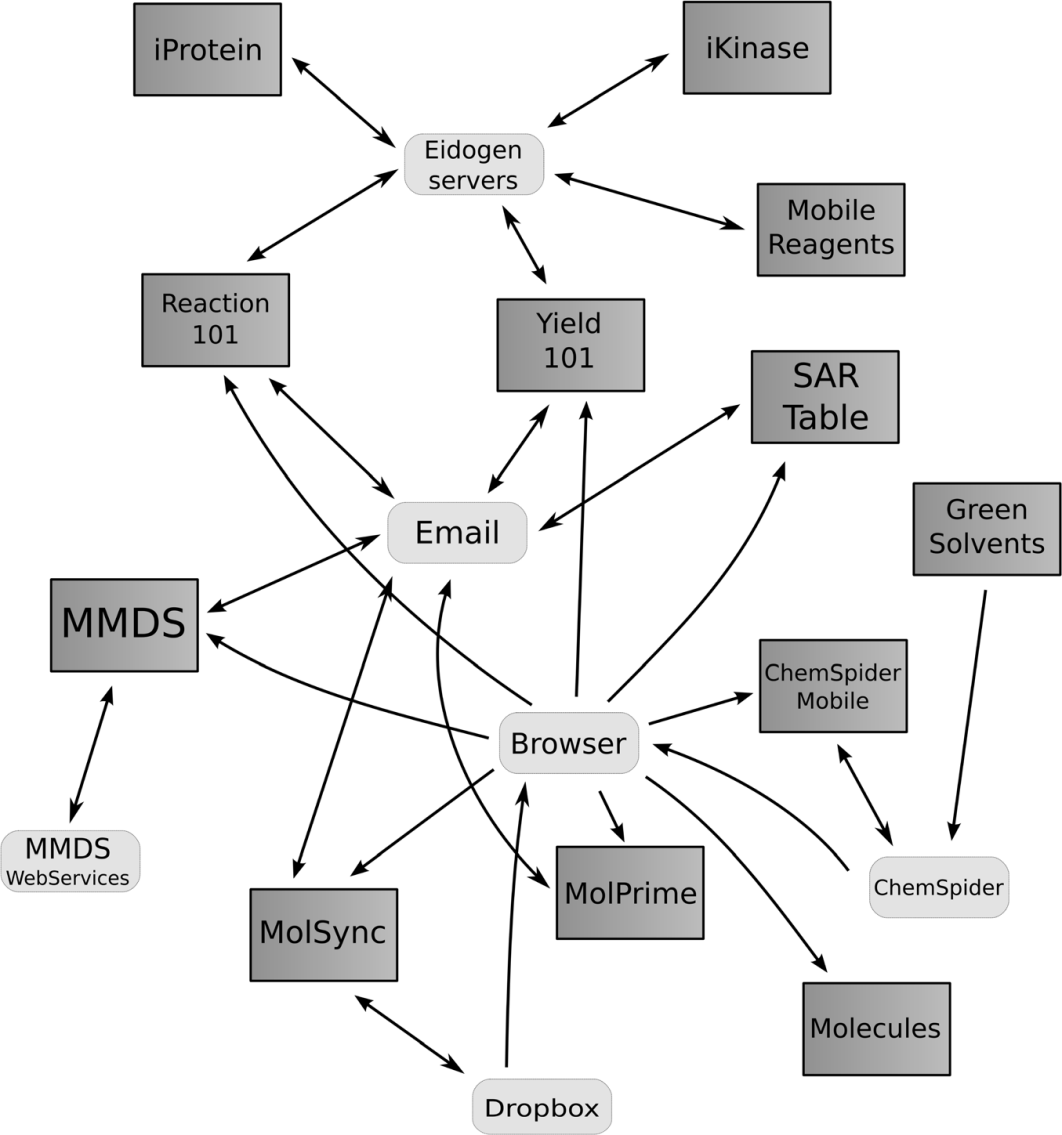
Interaction chart showing app capabilities for sharing data using networked services.

**Figure 2:**
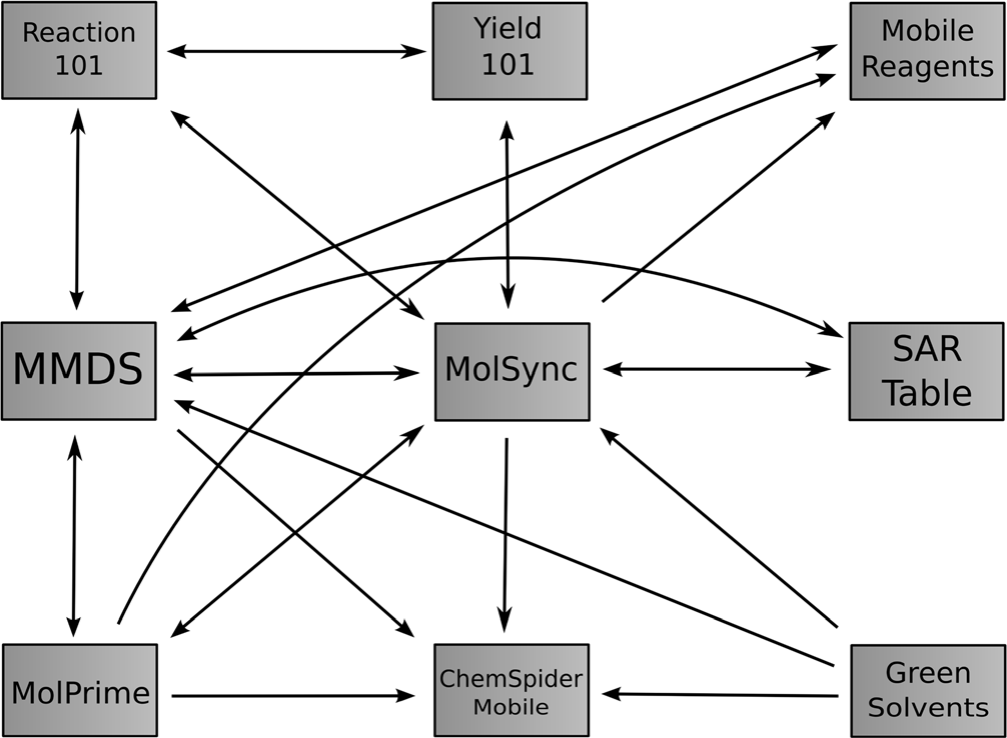
Interaction chart showing apps which can communicate directly by passing data using interprocess communication.

Currently available apps that are applicable to this discussion are those which deal with cheminformatics representations of chemical structures, either 2D or 3D, and have some ability to send or receive chemical data. Figure 1 shows an interaction chart indicating some of the communication pathways by which these apps are able to communicate using network protocols, while Figure 2 shows the interactivity from app-to-app using interprocess communication methods. Selected examples are shown in Table 1, each with a brief description of its functionality, while Table 2 summarizes the communication capabilities of these apps.

**Table 1:** The apps used as examples in this article.

Icon	App name and description
**Mobile Molecular DataSheet** (MMDS).^71^ A multifunctional chemistry app, which provides editing tools for structures, reactions and collections with auxiliary data. Supports all of the communication techniques described in this article.
**MolSync.**^72^ Chemistry-aware client for remotely stored files hosted by Dropbox. Provides viewing and editing capabilities, as well as serving as an intermediary for sharing content with other apps. Can be used to explicitly synchronize data with MMDS.
**SAR Table.**^73^ Specialized editor for building tables containing an homologous series of scaffolds and substituents, creating composite structures, and providing activity data. Can be used to prepare figures for manuscripts, or to recreate data from the literature.
**Reaction101.**^74^ Education-focused app which provides a reaction editor, with access to structure searching capabilities and a list of template reactions to use as a starting point. Provides an automatic stoichiometry calculator, and cloud-based storage.
**Yield101.**^75^ Lab-support app which allows quantities to be added to a reaction. Interdependent quantities can be calculated automatically, making use of stoichiometry and structure, to ultimately derive the reaction yield. Also calculates process mass intensity.
**MolPrime.**^76^ Lightweight app which provides structure editing, simple property calculation, and the launching of other services and features.
**Green Solvents.**^77^ **A reference app which compiles a list of common solvents with information about their environmental impact and hazards.**^78^ Content can be viewed within the app, or shared.
**ChemSpider Mobile.**^79^ A mobile client for the *ChemSpider* service that provides structure drawing capabilities. Once located, entries are viewed by launching the mobile web browser to open the mobile-friendly result page.
**Mobile Reagents.**^80^ A mobile access point to a large collection of commercially available compounds, with associated vendor information. Searches can be done by name, by drawing structures, or by photographing structures or QR codes.
**iProtein.**^81^ Mobile access to protein structures organized by sequence, structure/model, site, protein family and cofactors. Can be searched by name or small molecule structure. 2D ligands, 3D active sites and other information can be browsed.
**iKinase.**^82^ Provides access to the curated Kinase KnowledgeBase (KKB).^83^ which makes available structure activity data from throughout the kinome.
**Molecules.**^84^ 3D viewing capabilities for large biological molecules from the Protein DataBank. A variety of viewing modes and gestures are supported.

**Table 2:** Data sharing capabilities of selected apps. IPC=interprocess communication, RPC=remote procedure call.

App name	Chemical data	Communication methods
	Molecules	Reactions	Collections	Clipboard	Attachment	IPC	Hosted	RPC
MMDS	yes	yes	yes	in/out	in/out	in/out	in/out	in/out
MolSync	yes	yes	yes	in/out	in/out	in/out	in/out	in
SAR Table	–	–	yes	in/out	in/out	in/out	in/out	in
Reaction101	–	yes	–	in/out	in/out	in/out	in/out	in
Yield101	–	yes	–	in/out	in/out	in/out	in/out	in
MolPrime	yes	–	–	in/out	in/out	in/out	–	in
ChemSpider Mobile	yes	–	–	in/out	in	in	–	in
Green Solvents	yes	–	yes	–	–	out	–	–
Mobile Reagents	yes	–	–	in/out	in/out	in/out	–	in
iProtein	yes	–	yes	in/out	in/out	–	–	in
iKinase	yes	–	yes	–	–	–	–	in
Molecules	yes	–	–	–	in	–	–	in

The capabilities of apps to handle chemical data types is broadly classified into three types: molecules, reactions and chemical data. *Molecule* data refers to the ability to render and manipulate connection-table formats, such as MDL Molfile,^44^ SketchEl,^46^ PDB,^47^ etc. *Reaction* data refers to datastructures which group some number of molecules into reactants and products. *Collection* data refers to the ability to handle more than one molecule or reaction datum, along with auxiliary data such as text and numeric content. *Communication methods* refers to the ability of the app to pass data back and forth using the specified mechanism. *Clipboard* data sharing refers to being able to place machine-readable chemical data onto the system clipboard, for any other app to make use of, and to be able to read back such data from any app that uses a known format. *Attachment* data sharing refers to the app registering itself to be able to handle certain file types. This allows data to be imported from sources such as email attachments and web downloads, or to export data by methods such as initiating an outgoing email message containing attachments with chemical data. *Hosted* refers to the app being able to make use of chemical data that resides on a networked server, in a manner somewhat analogous to storing the data on the device itself. *IPC* (interprocess communication) refers to the ability to exchange chemical data with other apps on the same device, while *RPC* (remote procedure call) refers to the act of accessing a networked resource for some purpose, such as looking up data or invoking a calculation.

Many of the apps selected to illustrate the techniques described in this article make use of a common library, *MMDSLib*,^48^ which is at the core of the *MMDS* app. This library provides baseline cheminformatics functionality, e.g. molecule, reaction and datasheet handling, format interconversion, and reusable user interface components for molecule and reaction editing, and provides an alternative to re-implementing all of the necessary client-side functionality for each new app.

### 2.3 Clipboard Data Sharing

Almost all modern computing platforms, both desktop and mobile, provide a concept that is commonly referred to as the *clipboard* (or *pasteboard*, on the iOS platform) which is a global resource that can be accessed by any application. While support for complex datatypes varies considerably, the lowest common denominator is the ability to store a text string. In order to make chemical data available to *any* other app that requests the information, all that is necessary is to serialize the data object as a text representation, and invoke the appropriate API call to place it onto the clipboard. There is, however, often more than one compelling choice of format.

Consider the placement of a 2D structure of methanol onto the clipboard. If the largest possible range of possible recipients is desired, then the MDL Molfile^44^ format is a good choice, the text for which is shown in Table 3a. The MDL Molfile format is very popular, but is functionally obsolete,^49^ and is not as prevalent with mobile apps as with desktop apps. Most of the apps considered in this article use the SketchEl format by default, shown in Table 3b, when copying structures to the clipboard, since it can be used to transmit structures without information loss.

**Table 3:** Placement of a molecular structure onto the clipboard as text using (a) MDL Molfile format and (b) SketchEl format.

(a)																						
	name
																						
	2	1	0	0	0	0	0	0	0	0999	V2000
	0.0000	3.1500	0.0000 C	0	0	0	0	0	0	0	0	0	0	0	0
	0.5000	3.1500	0.0000 C	0	0	0	0	0	0	0	0	0	0	0	0
	1	2	1	0	0	0	0															
	M END
	
(b)	SketchEl!(2,1)
	C=0.0000,3.1500;0,0,i3
	O=1.5000,3.1500;0,0,i1
	1–2=1,0
	!End

For some purposes, it is also useful to be able to copy a SMILES^50^ string onto the clipboard. One example is when using the browser in combination with a website that accepts SMILES strings as structure queries. This is not a suitable way for transmitting structures between apps, however, since all layout information is lost. For more complex data types, such as reactions, or datasheets, which can combine a variety of structures, reactions, data and metadata, there are a number of formats to choose from, but most of the apps use the XML datasheet format.^51^

When an app offers the *paste* feature for chemical data, it operates by examining the system clipboard, and attempting to parse the string content within, searching for any types which are appropriate to the context (e.g. molecules, reactions, datasheets, or plain text). This process is typically done by trial and error, i.e. continuing to attempt to parse applicable formats, until one succeeds, or none remain.

### 2.4 Attachment Handling

One of the most effective ways to import data into an app is to register it for handling specific file type attachments. Certain operations involve asking the operating system to select a receiver app for a particular file, which is identified either by its file extension or MIME type. Key examples include those in Table 4. For the iOS platform, MIME types are used as the primary identifier for file type when the mobile browser is directed to a file that it cannot handle internally. The webserver provides the MIME type, as well as the filename. Similarly for email attachments, the MIME type is an intrinsic property of the attachment.

**Table 4:** Molecule formats and MIME types.

Format	Ext	MIME type
MDL Molfile	.mol	chemical/x-mdl-molfile
SketchEl	.el	chemical/x-sketchel
XML DataSheet	.ds	chemical/x-datasheet
MDL SDfile	.sdf	chemical/x-mdl-sdfile
MDL RDfile	.rdf	chemical/x-mdl-rdfile
MDL RXNFile	.rxn	chemical/x-mdl-rxnfile
Chemical Markup Language	.cml	chemical/x-cml
ChemDraw	.cdx	chemical/x-cdx

When opening an attachment, the list of installed apps that have advertised their ability to handle the type is generated, and the user selects one. The app is then launched, if it is not already running, and sent an instruction to open the resource. The filename and the data are provided, for which the app must decide on a course of action.

The *MMDS* app can handle attachments composed of any of the data formats listed above. For Chemical Markup Language (CML)^52^,^53^ and ChemDraw^54^ content, it makes use of a remote procedure call to convert it to the XML datasheet format. All other incoming data is parsed by the app itself. Incoming data can be resolved into three different flavors of chemical data: a single molecular structure, a single reaction, or a tabular datasheet containing any number of molecules, reactions and data. The incoming data is added to the content store locally by *MMDS*, allowing the user to make use of it in the same way as content that was created using the app itself. *MolSync* offers the same inclusive set of attachment data formats, but it differs in its response: rather than storing the data locally, it initiates the process of uploading the data to a remote data repository.

The *Reaction101* and *Yield101* apps register themselves as attachment handlers for reaction-type data, which allows the attachment handling mechanism to define the current reaction, while the *SAR Table* app ideally expects to receive a datasheet that conforms to its own tabular layout constraints, but will also accept other data collections, such as MDL SDfiles. Other apps, such as *MolPrime*, *ChemSpider Mobile*, *Mobile Reagents*, *iProtein* and *Molecules* can be opened using attachments that describe a molecular entity, which can be viewed and used.

### 2.5 Interprocess Communication

Communication between two apps installed on the same iOS-based device is possible via a mechanism that is closely related to the attachment handling process. While the platform itself keeps apps strictly separate, there are two ways to initiate the transfer of data to another app

1 Create a file in the app′s private directory, and instruct the operating system to open it.
2 Define a custom URL scheme to identify a specific app.

The first technique is generic, and involves handing over control to the operating system. For example, creating a file in the working directory called something.mol, then invoking the *open-in* functionality, builds a list of installed apps that advertize their ability to open. mol files. The list is presented to the user, and if one is selected, that app is launched, in the same fashion as for opening an attachment. The second technique is specific, and requires coordination between the apps involved. In addition to file extensions and MIME types, an app can define a custom URL prefix. For example, *MMDS* defines the mmds:// prefix, which means that URLs can be constructed in order to transmit messages. While messages passed via the URL string are necessarily short, it is possible to combine this functionality with a named pasteboard in order to deliver a data payload. When the custom URL scheme is invoked, the target app will be launched, and will receive the URL, and have an opportunity to examine the data from the named pasteboard.

For example, *MMDS* can be invoked by other apps using the generic mechanism, because it is registered to handle file extensions such as. mol and. el, but if another app wants to ensure that *MMDS* is the recipient of interprocess communication, this can be accomplished by acquiring a new named pasteboard, e.g. “moldata”, and setting it with an MDL Molfile-formatted string. Then the *MMDS* app can be invoked by opening the URL

mmds://pasteboard?src=moldata&type=chemical/x-mdl-molfile&action=open

Besides being able to specifically indicate the destination of the interprocess communication, it is also possible to find out whether the app is installed, by querying whether or not the URL can be opened. This is particularly useful when building apps with the explicit intention of having them work together: if a companion app is not installed, it is a simple matter to suggest that the user may wish to visit the AppStore to acquire it, which is in contrast to the generic method, for which missing apps are omitted from the list.

### 2.6 Hosted Data Access

Making general-purpose HTTP requests from a mobile app is straightforward, and is a very important mechanism by which apps provide functionality that extends beyond the limited capabilities of the device. There are many good reasons to use a network transport mechanism to store data on a centralized server other than the device itself, which include data protection and opportunities to share the data between other apps, other devices and colleagues. For iOS-based apps, which are unusually heavily sandboxed and have no commonly accessible file system, storing data on remote servers is often used as a way to pass data between apps.

Many apps are associated with custom data hosting services, while others implement documented protocols. For example, the *Reaction101* and *Yield101* apps both store the current reaction scheme on the device itself, but provide a way to upload schemes into a *personal* collection that is hosted by cloud-based servers maintained by Eidogen-Sertanty.^55^ The data is associated with the device, which allows both of these apps to share the same content.

A much finer degree of control is provided by the *MolSync* app, which is effectively a chemically-aware shell for the *Dropbox* service. The app makes use of the documented API for connecting a user to an existing account and navigating the file system. Files with chemical content are recognized, and value added services are provided, e.g. viewing, editing, translating file formats, and producing graphics from chemical data. Because the *Dropbox* service is broadly accessible from essentially any internet-capable device, it is a relatively direct form of communication: data can be uploaded from an alternate source and accessed immediately from a mobile device, and likewise data created on a mobile device can be made immediately available to other platforms.

Combining remote file hosting with specific inter-process communication is an example of how data sharing makes individual apps considerably more powerful when used in combination: *MolSync* and *MMDS* are designed to work together in concert. Both apps recognize special metadata which is stored within datasheet files. These files can be used to establish a correspondence between data stored by *MMDS* on the local storage of the device, and the remote file location that is accessed by *MolSync*. The two apps communicate by interprocess communication, which allows datasheets to be modified on the device and refreshed on the remote file system, or updated on the device if they have been modified by another agent.

The metadata is designed in such a way that any number of devices can be associated with the file. If multiple users are sharing the same *Dropbox* account, or if the folder-sharing features of *Dropbox* are used, this capability is transformed into a collaboration tool, simply by leveraging capabilities that are provided by a third party service.

For purposes of sharing data on the internet, it is necessary to store it in a centralized location where it can be accessed with a URL. While there are innumerable ways to accomplish this, one very convenient way is to upload chemical data to a specially designated *public* folder, which is a standard feature of all *Dropbox* accounts. The *MolSync* app recognizes the special properties of this folder, and can formulate a URL which leads directly to the content. From within the app, this content can be opened within the browser by directing access to the raw data through a website which renders it using HTML5 capabilities, as well as offering access to cheminformatics functionality, such as transcribing the data into a variety of different formats.^56^ This feature is just one of many ways for making data publicly available, and is one of the most convenient ways to accomplish this from directly within a mobile app. The ability to link mobile-created chemical data to a URL that is available to anyone also provides a viable entry point into the realm of sharing via social networking, and in fact publicly shared data can be “tweeted” directly from within the *MolSync* app, by making use of the *Twitter* integration features that were introduced to iOS 5.

Access to *Dropbox* and *Twitter* posting is carried out via secure HTTP, and uses username/password credentials to establish an active session and, as a result, these services can be considered somewhat secure.

### 2.7 Remote Procedure Calls

Use of the HTTP network transport mechanism is the central technology for allowing mobile apps real-time access to functionality that is not provided by the app itself. There are a number of pragmatic and strategic reasons for offloading functionality to an external service. Chemistry apps that are currently in use make use of network resources for two main purposes

1 Performing calculations that are not provided by the app itself.
2 Accessing centralized data resources.

While mobile devices have enjoyed a steep rise in computational capacity in recent years, they will never be able to match a stationary computing resource - nor should they, since battery life is an important issue. Software development tools are also an important consideration, since mobile devices typically offer only a narrow subset of programming languages and class libraries, while a cloud-hosted server typically has few such limitations. Perhaps most importantly, much chemistry-oriented functionality relies on access to data repositories which are far too large to store on a remote device, and are regularly updated in a centralized location.

The range and diversity of remote procedure calls for currently available chemistry apps is large, and growing fast. *MMDS, MolSync* and *SAR Table* apps make use of a cloud-based server to offload some of the more complex file format conversions and presentation-quality graphics output to a remote server. The *ChemSpider Mobile* and *Mobile Reagents* apps exist primarily to provide an interface to a centralized database of small molecules, which are accessed by a web services interface. The *Reaction101* and *Yield101* apps can be used as standalone apps without network connectivity, but they are augmented by their ability to store personal content on a cloud-based server, and provide integrated access to the same data content used by *Mobile Reagents*. Both *iProtein* and *iKinase* provide access to high-value curated data, which is made available by a web services interface, and the *Molecules* app provides access to the Protein Data Bank.^57^

In consideration of the design of web services for providing remote procedure call functionality, the most convenient protocols are those using plain XML or JSON/RESTful styles, since messages can be composed and parsed by the client using a simple API. More complex protocols, such as WSDL/SOAP, incur a greater burden on the client. The remote procedure calls described in this article operate on the assumption that the service will finish execution after a short timeout (e.g. 30 seconds), after which time the request will be considered to have failed. Operations which require more time can be decomposed into multiple requests, e.g. establishing a session context, then polling for partial or complete results. The remote procedure call services described in this article do not require login credentials and use the standard HTTP transport mechanism. If security is an issue, they should be hosted within a private intranet.

### 2.8 Custom web Services

The *MMDS* app is currently unique in that it includes a generalized method for accessing web services, as well as fixed-purpose remote procedure calls. *MMDS* web services make use of a simple and openly documented protocol^58^ which operates according to the following workflow

1 Download a directory of available services, and store these in a cached list.
2 When a service is used for the first time, or refreshed, download the parameter specification.
3 Present the parameters to the user in the form of a dialog view, allowing each value to be specified.
4 On execution, send the values for each of the parameters to the web service.
5 The web services performs its task, and returns a data collection, in the form of an XML datasheet or MDL SDfile.
6 The resulting data collection is added to the locally-stored content, and can be viewed and manipulated.

The set of parameters that a web service may require is composed of molecules, numbers, text, flags, option lists and datasheets. The chemical datatypes – molecules and datasheets – can be provided using the editing features of the app.

At the time of writing, example web services include search clients for both *PubChem*^59^,^60^ and *ChEBI*,^61^ as well as a melting point calculation service.^62^ Because the protocol is general and open, and documented with source code examples, it is relatively straightforward to interface any existing cheminformatics functionality with the app, without having to make any modifications to the client.

## 3 Results

**Figure 3:**
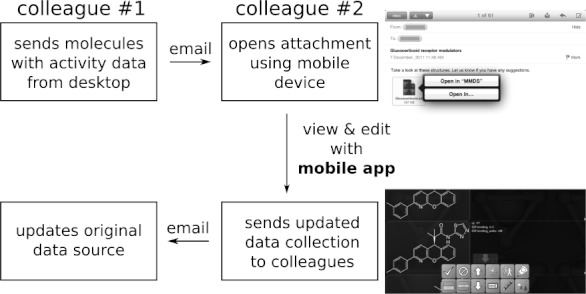
Use of *MMDS* to share collections of structure/activity data between two colleagues, one using desktop software, and the other using a mobile device.

There are numerous practical scenarios where chemistry-enabled mobile devices can be inserted into a workflow in which chemical data is communicated. For instance, the *Mobile Molecular DataSheet* (referred to as *MMDS*) was the first mobile app to integrate a powerful sketcher^63^ with data management and communication capabilities. Figure 3 illustrates the following workflow: if a member of a research team is away from the laboratory but still working, e.g. at a conference, a colleague may be assembling a collection of compounds with measured activity values against a drug target, using desktop software of choice or by downloading the collection of data from an online database. By exporting the data collection to a standard format, e.g. MDL SDfile, and sending it by email, the absentee colleague can use a BlackBerry or Apple mobile device to extract the email attachment using the *MMDS* app.

Once the app has parsed the attachment, it becomes a viewable and editable *datasheet* that is stored on the device. Any member of the research team who received the email can then use the app to study the content, and make suggestions, e.g. editing, adding or deleting structures, activities, names, auxiliary data, etc. Once any changes are applied, they can be sent back as email attachments. This is a much more efficient workflow than crude alternatives using generic technology, e.g. sending the data as a PDF, and requiring any suggestions to be mailed back in plain text, or explained during a phone call, and manually applied to the data at its point of origin.

**Figure 4:**
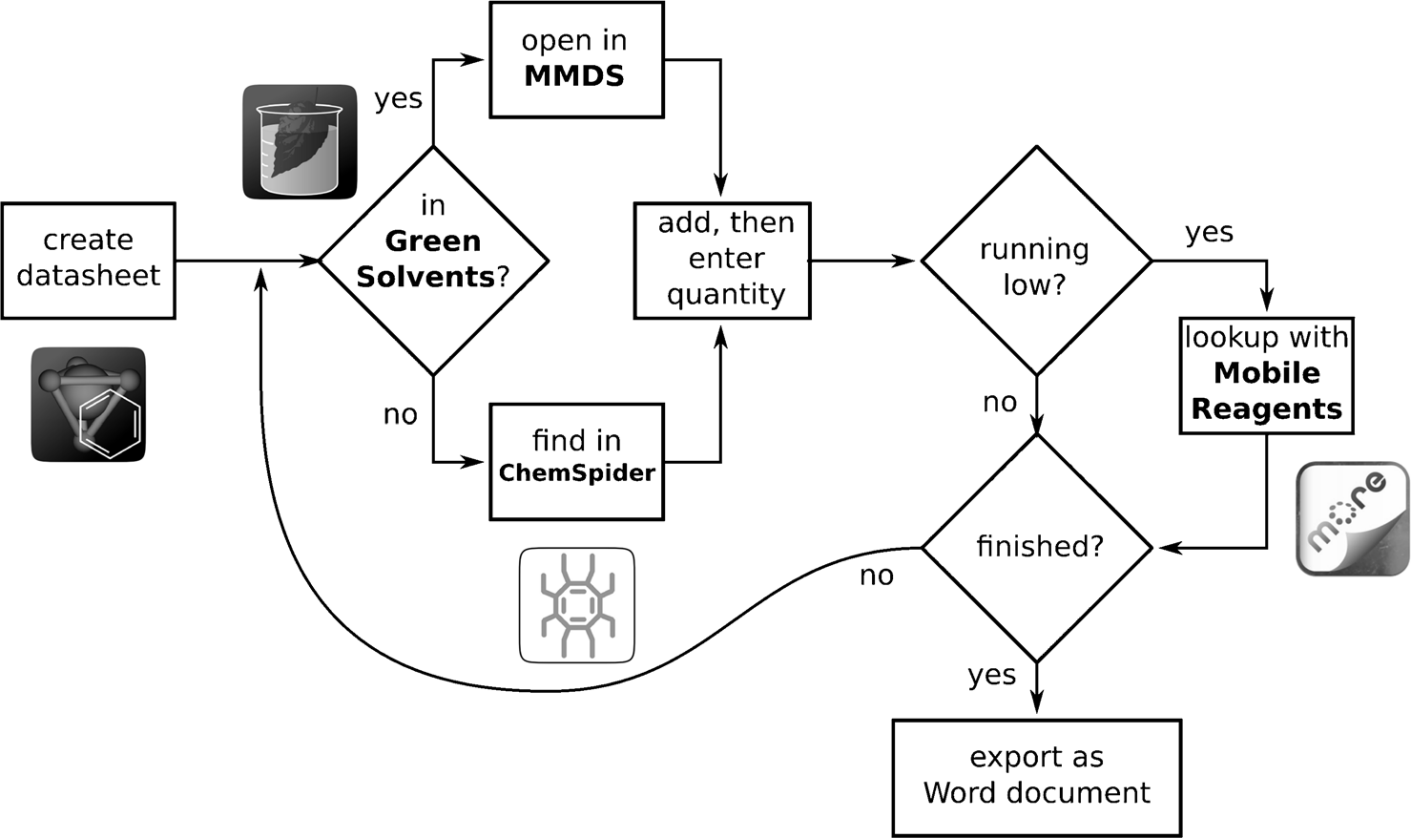
Use of four apps to compile an inventory of solvents: *MMDS*, *Green Solvents*, *ChemSpider Mobile* and *Mobile Reagents*.

Besides interacting with colleagues through a network using industry standard data formats, the process of building up a datasheet with structures and auxiliary data can be aided by intra-app communication. Figure 4 illustrates a multi-app scenario: consider a laboratory-wide inventory of solvents that are currently on hand, where each solvent needs to be represented by structure, name and quantity. Being able to enter this data on a mobile device has significant convenience value, since solvent cabinets are usually not located within reading distance of a conventional workstation. The *MMDS* provides enough functionality to carry out this task, but the process can be streamlined by making use of supporting apps.

As each solvent bottle is examined, the *Green Solvents* app can be quickly consulted to see if it is in the list. If so, the name, structure and environmental properties can be transferred to *MMDS* via interprocess communication. If the solvent is not in the list, but the name or structure is clearly indicated on the bottle, it can easily be looked up using the name search capabilities of the *ChemSpider Mobile* app. Once the material is located, the mobile browser can be opened to the corresponding ChemSpider page for the entry, which provides a wealth of information about the compound and links out to a potential of hundreds of original data source websites on the internet. It also allows the 2D structure of the compound to be downloaded, as an MDL Molfile. Downloading a file with a chemical data format triggers a matching of the format and app capabilities: the structure can be opened using *MMDS*, and added to the locally stored collection. Similarly, if a solvent has almost run out, it might instead be looked up using the *Mobile Reagents* app, which offers a variety of additional search types. Once the material is located, various additional information is provided regarding the suppliers, which is relevant for preparing a purchase order. The *Mobile Reagents* app is also capable of sharing data directly with *MMDS* via inter-process communication, so the resulting structure can be added to the solvents datasheet internally on the device, rather than via a remote download. The *Mobile Reagents* app can also make use of the device camera to scan QR codes^64^ to look up the compounds, which introduces new possibilities for a solvent labeling system.

Once the collection of solvents is complete, it can be shared with any number of colleagues, and as long as they have software that can edit industry standard formats such as MDL SDfile, either on desktop or mobile devices, they can make edits and pass them back. When the datasheet is ready, it can be converted into a presentation-ready format, such as a Microsoft Word document, using a remote procedure call between the mobile device and a hosted service. The resulting document can be exported by email, and unpacked on a workstation, so that final modifications can be made before printing and distribution of the solvent list.

Mobile devices can also be used to handle chemical reactions as well as structures of single molecules and associated data. It is possible to draw and balance a chemical reaction on a mobile device using the *Reaction101* app. It is also integrated with the same web service developed for the *Mobile Reagents* app, which allows individual reaction components to be looked up by searching the database directly, without having to switch to a different app.

**Figure 5:**
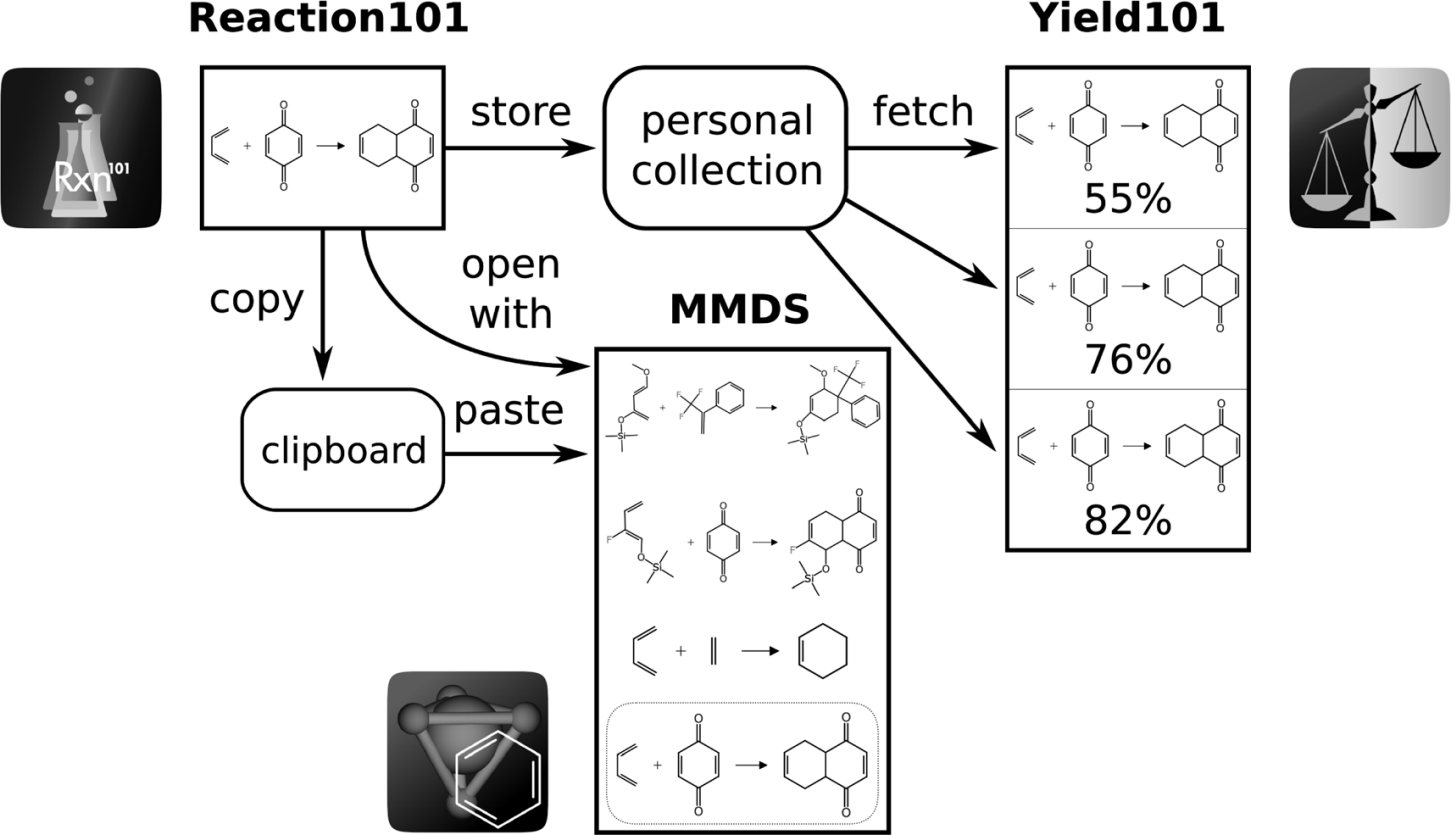
Drawing of chemical reaction diagrams using *Reaction101*, sharing with *Yield101* to create quantitative schemes, and using *MMDS* to gather reactions into a collection.

Once the reaction is specified, it can be uploaded and stored as a personal collection on a remote server, which is associated with the device, so it can be recalled later. It can also be recalled by its companion app, *Yield101*, which can use pre-drawn reactions as a template. These capabilities are illustrated in Figure 5. This approach thereby facilitates data sharing via a hosted server. The *Yield101* app adds quantity fields which are calculated automatically whenever they are implied. This makes it very useful for describing a single experimental instance of the reaction. Quantities can be provided as soon as they are known, and this is a good reason to keep a mobile device handy at the lab bench. The calculated quantities can be transcribed into a paper lab book, or a report of the reaction, with provided and calculated amounts, can be prepared as a PDF file, and either transmitted by email or sent directly to a printer from the device itself.

While *Reaction101* provides the most convenient way to specify individual reactions, *MMDS* provides the ability to gather them together in datasheets, and associate them with additional information. Reactions can be transferred from the *Reaction101* app by a number of techniques: by copying to the clipboard, by network transfer by sending an email to oneself, or by invoking the *Open with* feature and selecting *MMDS* as a destination app. In this way, datasheets containing a collection of reactions can be built up, by using either of these two apps to draw individual reactions.

**Figure 6:**
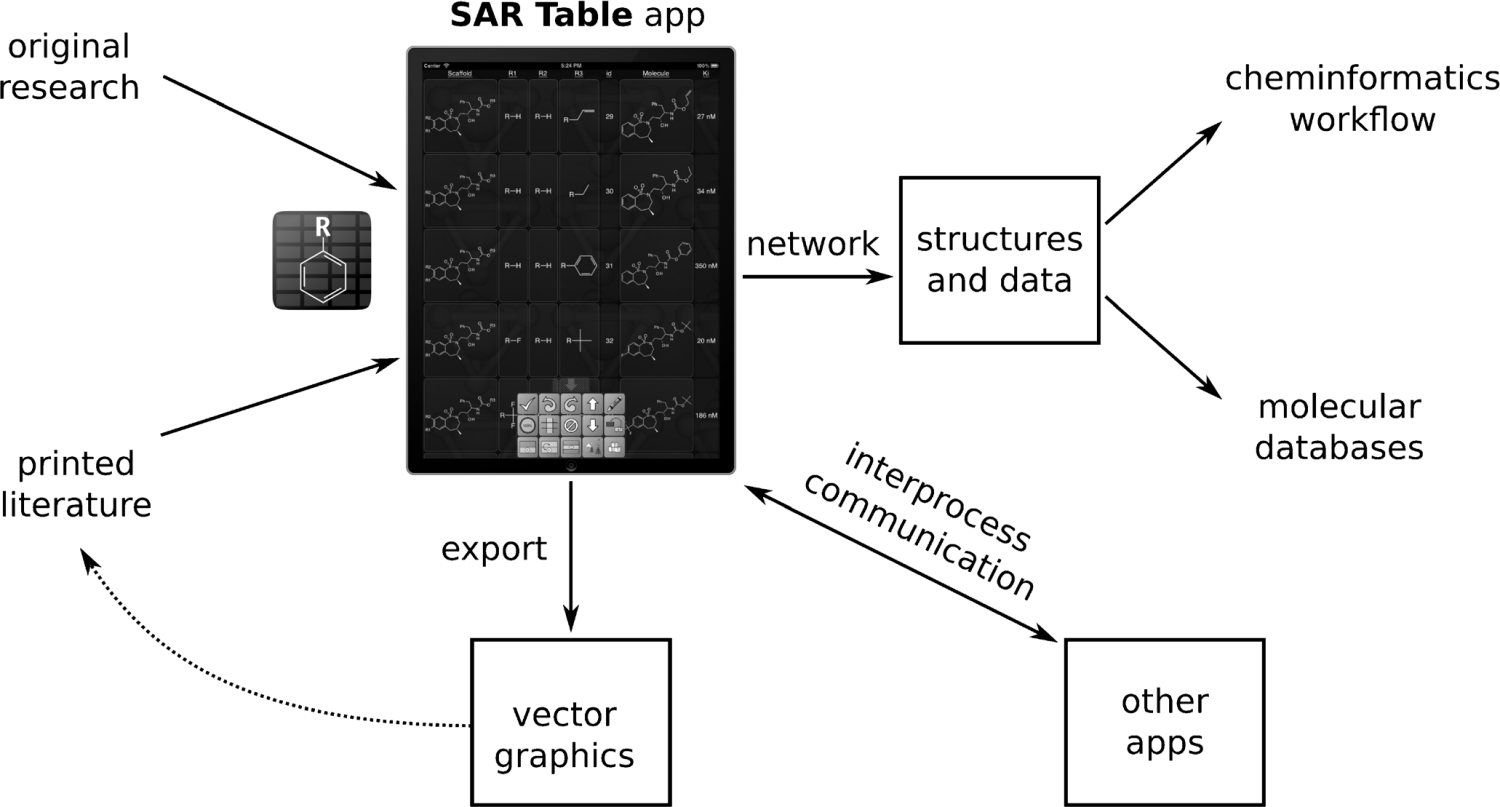
The *SAR Table* app is used to provide structure and activity data for an original or existing series, which can be used to generate publication quality graphics, to feed into a cheminformatics workflow, or exchange data with other apps.

One common task that occupies a significant amount of time for computational chemists is the extraction of chemical structures from publications with tables encoding structure-activity relationships, which often use a highly abbreviated and compact form.^65^–^57^ The *SAR Table* app was designed to reduce the amount of repetitive structure redrawing required to recreate this data, in a tabular form, which includes Markush-style scaffolds, substituents, the structure of the fully assembled molecule, along with activity data and any relevant text or identifiers. The workflow capabilities of the app are illustrated in Figure 6. The *SAR Table* app can conveniently be used away from the desk, e.g. at a library, or while travelling with a printed hard copy of the publication from which the data is being entered. Once the table is complete, it can be exported via email, in any number of file formats, which can be used for modeling studies such as fingerprint analysis^68^ or QSAR,^69^ or submitted to conformational analysis and used with various 3D techniques.^68^ The app can also be used to create such structure activity tables for publication purposes: the resulting data can be exported via email, using pre-print file formats such as a Microsoft Word document^70^ with an embedded table containing vector graphics for each of the structure fragments. The file can then be modified using the aforementioned word processing package, and integrated into a manuscript.

**Figure 7:**
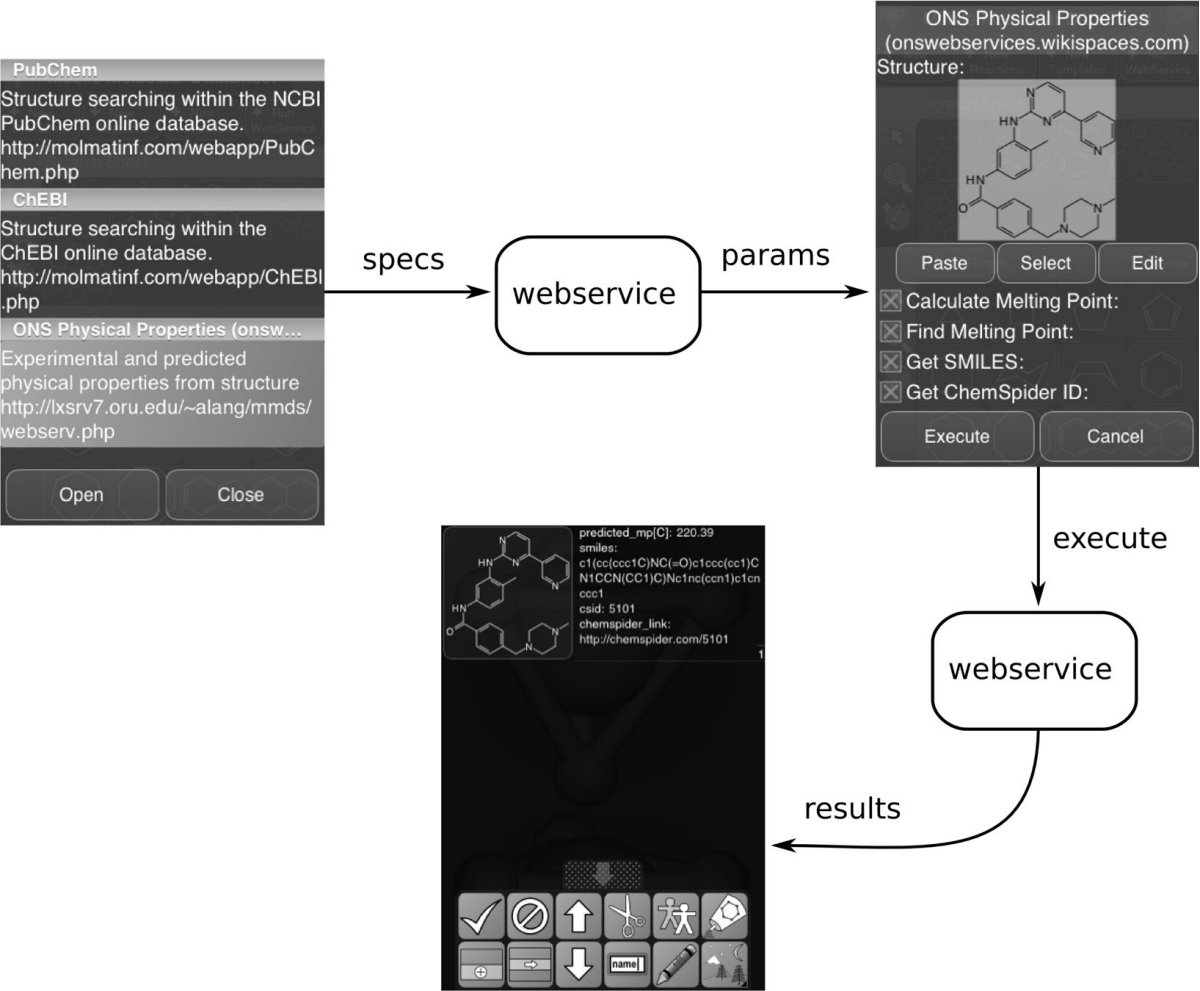
Invocation of the *Open Notebook Science* melting point calculation service, and viewing of the results.

While a number of apps exist with the primary purpose of making data available to the user, the multifunctional *MMDS* app includes a general purpose web services access point that can be used to discover custom-designed web services. The web service itself specifies the form of the input, and the app produces a dialog form, with which the user can provide the required data (e.g. molecules, datasheets, numbers, text, flags and option lists). The default services currently include search capabilities for *PubChem*^60^ and *ChEBI*,^61^ as well as submission of molecular structures for melting point calculations,^62^ which is illustrated by the example shown in Figure 7. Because the web service functionality is general and based on an open protocol, it is possible to build customized services to complement the standard list.

**Figure 8:**
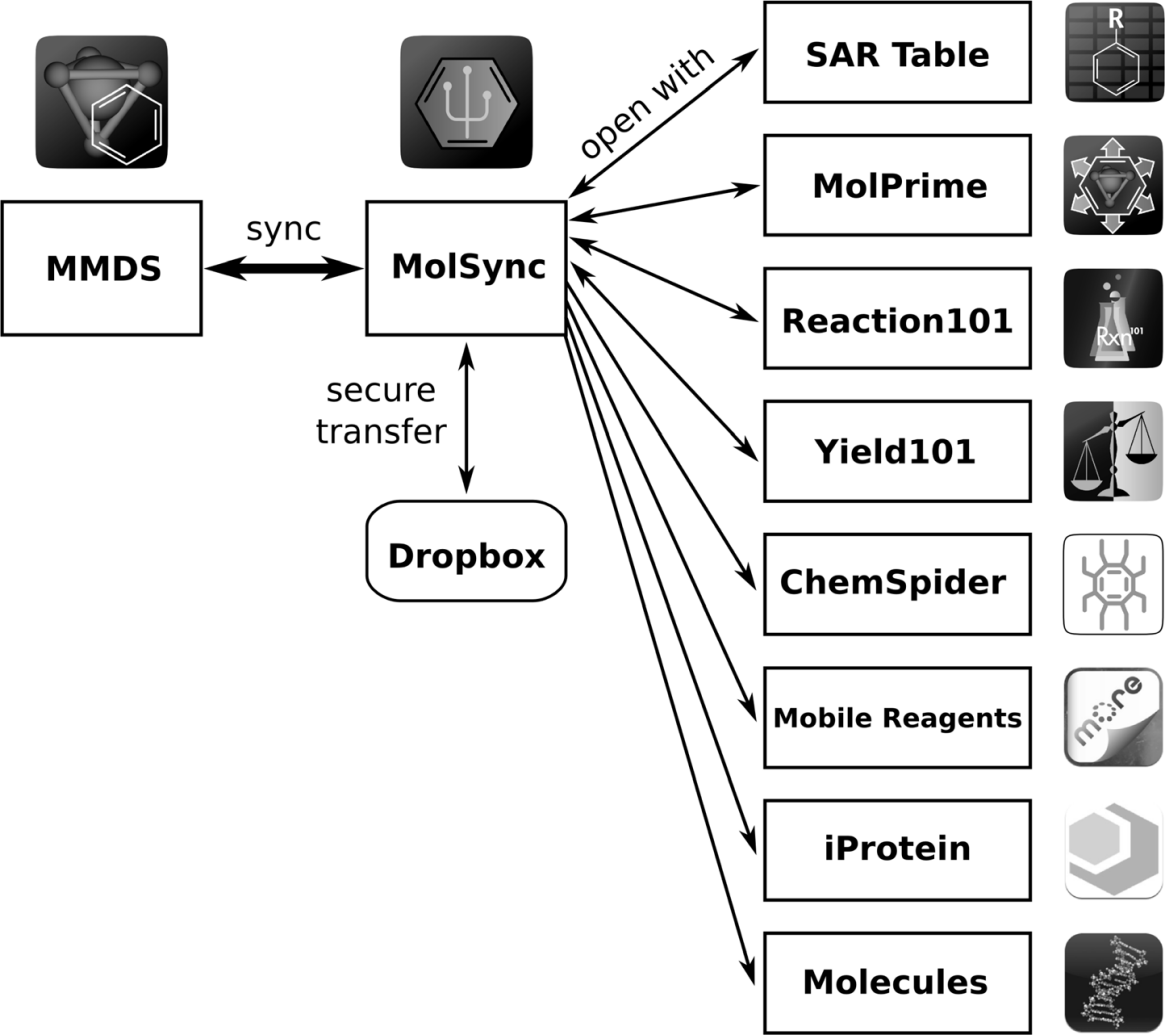
The role of *MolSync,* as the intermediary app, making remote content stored on *Dropbox* available to other apps via interprocess communication.

A datasheet that is maintained within *MMDS* can be stored on a remote file hosting service using a successive chain of technologies. If the *MolSync* app has been installed and configured, then it will already be connected to a *Dropbox* account.^45^ The *MMDS* app can use interprocess communication to instruct *MolSync* to *synchronize* a datasheet with an online repository, which is illustrated in Figure 8. This process involves annotating the datasheet with extra metadata which tracks the correspondence: the file will be uploaded to *Dropbox*, and it will be associated with the datasheet that is stored within the collection of data stored on the mobile device, within the private file area of the *MMDS* app. If the file is updated using *MMDS*, or updated on the *Dropbox* repository, the two can be re-synchronized, in order to update the changes.

The remotely stored datasheet can be associated with more than one instance of *MMDS*, which is particularly useful for users who have more than one mobile device (e.g. a phone and a tablet), which can be easily kept up to date. Making use of sharing features that are provided by *Dropbox* enables this to become a collaborative tool: by sharing a file or a folder with another user of *Dropbox*, who also uses *MMDS* on a mobile device, both users can re-synchronize local copies of the same datasheet to send or receive modifications.

It is also possible to make content publicly available using this process. Because *Dropbox* provides a special public folder, it is possible to construct a URL that can be used to download any file in this folder, without requiring any access credentials. Using this feature, *MolSync* provides a built-in tweeting capability for chemical data stored in the public folder. The newly instantiated tweet is given a link which acquires the chemical data and presents it graphically on a web page, and allows it to be downloaded in a variety of chemical data and graphics formats.

## 4 Conclusions

The pace at which mobile apps have claimed a prominent position within the workflow of so many professionals is impressive. To a large extent this transformation has been spearheaded by the Apple iPhone, which was only released in June 2007, while the iPad was released in January 2010. Considering the penetration these app-oriented mobile devices have accomplished in such a short time, and the traction of the recently announced Android development environment, it is clear that we are only in the early stages of this new mobile technology surge. The new generation of scientists was born as digital natives and will expect increasing performance and capabilities from their mobile devices. Already the capabilities of mobile devices to access, search, manipulate and exchange chemistry-related data almost parallel those capabilities which were available on desktop computers just a few years ago. This article represents the present state-of-the-art in terms of apps for dealing with chemical compounds and reactions and especially in terms of data exchange for the purpose of collaboration. We are confident that this budding ecosystem of chemistry apps will continue to grow rapidly, and that the ability of these apps to complement each other, as well as workstation-based and server-based software, will secure their place within chemical data workflows.

Second generation cheminformatics apps will have the facility to perform many more sophisticated functions, and in order to make ever more powerful functionality practical, these apps will need to incorporate data sharing and collaboration features as an integral part of their design. There are numerous examples of cheminformatics and molecular modeling tools that we can imagine, or have already begun to design, as mobile apps, e.g. QSAR data preparation and prediction, pharmacophores, docking clients, 2D depiction tools for 3D data, to name but a few. Numerous additional data sharing scenarios are possible, e.g. deeper integration with online chemical databases, direct integration with electronic lab notebooks and interfacing with laboratory instrumentation via wireless communication methods such as Bluetooth. The combination of a user interface designed and optimized for the mobile form factor, cloud-based server functionality for data warehousing and extra computational capacity, and collaboration features for integration into an overall workflow, makes these projects not only technologically feasible, but in many ways preferable to traditional software.

Mobile apps are much less well suited to managing big data collections than analogous desktop software, due in large part to their limited computational and storage resources. Because apps function as components, frequent data sharing is a necessary part of any workflow, which is effective for small collections, i.e. hundreds of rows of data, rather than thousands or millions. Simple workflows involving big data collections, e.g. submitting a structure search to a server and fetching the best few results, are already well established. Active participation in visualization and maintenance of large data collections will require new methods for task subdivision and integration of apps within pipeline-based workflows.

The increased availability of data and algorithms in the cloud, accessible via standard programming interfaces, enables the first generation of scientific apps to access capabilities that will satiate many of the basic software needs of a practicing chemist. A recent review by the authors has described our experiences in terms of what mobile platforms can presently provide to the drug discovery chemist as well as what may be possible in the future.^42^ It is our contention that these apps will have impact across chemistry related industries and in the classroom both at high school and university levels.^64^ Following on from the development of a powerful yet intuitive system for sketching molecular structures on a mobile device,^63^ this article discussed one of the most crucial features for making mobile devices a viable component of a chemistry workflow, which is the ability to collaboratively share chemical data. A second generation of mobile apps is already emerging, which takes advantage of the many different technologies provided by mobile platforms that allow data to be passed back and forth between heterogeneous environments. This development is leading to the creation of a new ecosystem of chemistry-aware mobile apps, each of which has access to a variety of ways to share data with other apps on the same device, share data across the internet with other users, and provide access to a rich tapestry of internet chemistry resources.

## Abbreviations

MMDS Mobile Molecular DataSheet
ADME/Tox Absorption, Distribution, Metabolism, Excretion/Toxicity
SAR Structure – Activity Relationships
QSAR Quantitative Structure – Activity Relationships
HTTP Hypertext Transfer Protocol
URL Uniform Resource Locators
MIME Multipurpose Internet Mail Extensions

## Conflicts of Interest

Antony J. Williams is employed by The Royal Society of Chemistry which produces ChemSpider and ChemSpider Synthetic Pages mobile apps discussed in this article. Sean Ekins consults for Collaborative Drug Discovery, Inc. Alex M. Clark is the owner of Mobile Molecular Informatics, Inc., which has produced many of the apps described in this article.
